# Community perceptions of a malaria vaccine in the Kintampo districts of Ghana

**DOI:** 10.1186/1475-2875-12-156

**Published:** 2013-05-07

**Authors:** Lawrence G Febir, Kwaku P Asante, Dan-Bright S Dzorgbo, Kojo A Senah, Timothy S Letsa, Seth Owusu-Agyei

**Affiliations:** 1 Kintampo Health Research Centre, P. O. Box 200, Kintampo, Ghana; 2 Sociology Department, University of Ghana, PO Box LG 65, Legon, Ghana; 3 Regional Health Directorate, Ghana Health Service, PO Box 145, Sunyani, Ghana

**Keywords:** Vaccines, Malaria, Perceptions

## Abstract

**Background:**

Malaria remains the leading cause of morbidity and mortality in sub-Saharan Africa despite tools currently available for its control. Making malaria vaccine available for routine use will be a major hallmark, but its acceptance by community members and health professionals within the health system could pose considerable challenge as has been found with the introduction of polio vaccinations in parts of West Africa. Some of these challenges may not be expected since decisions people make are many a time driven by a complex myriad of perceptions. This paper reports knowledge and perceptions of community members in the Kintampo area of Ghana where malaria vaccine trials have been ongoing as part of the drive for the first-ever licensed malaria vaccine in the near future.

**Methods:**

Both qualitative and quantitative methods were used in the data collection processes. Women and men whose children were or were not involved in the malaria vaccine trial were invited to participate in focus group discussions (FGDs). Respondents, made up of heads of religious groupings in the study area, health care providers, traditional healers and traditional birth attendants, were also invited to participate in in-depth interviews (IDIs). A cross-sectional survey was conducted in communities where the malaria vaccine trial (Mal 047RTS,S) was carried out. In total, 12 FGDs, 15 IDIs and 466 household head interviews were conducted.

**Results:**

Knowledge about vaccines was widespread among participants. Respondents would like their children to be vaccinated against all childhood illnesses including malaria. Knowledge of the long existing routine vaccines was relatively high among respondents compared to hepatitis B and *Haemophilus influenza* type B vaccines that were introduced more recently in 2002. There was no clear religious belief or sociocultural practice that will serve as a possible barrier to the acceptance of a malaria vaccine.

**Conclusion:**

With the assumption that a malaria vaccine will be as efficacious as other EPI vaccines, community members in Central Ghana will accept and prefer malaria vaccine to malaria drugs as a malaria control tool. Beliefs and cultural practices as barriers to the acceptance of malaria vaccine were virtually unknown in the communities surveyed.

## Background

Malaria is still endemic in sub-Saharan Africa. *Plasmodium falciparum* malaria is estimated to cause millions of clinical episodes worldwide with majority in sub-Saharan Africa [1,2]. In Ghana, the burden of malaria is high with about 323 per 1,000 cases reported among children under five years of age in 2008; there has been some evidence of a decrease in malaria case in more recent years [3]. The burden of malaria in Ghana and other endemic countries leads to anaemia and cerebral malaria, resulting from severe or uncomplicated malaria if uncontrolled. Interventions such as rapid diagnosis, prompt appropriate treatment, intermittent preventive treatment of pregnant women with sulphadoxine-pyrimethamine, use of insecticide-treated nets (ITNs) and indoor residual spraying are beneficial and currently used in routine implementation as part of the strategies to control malaria.

These methods do not exist without challenges. Though ITNs are known to reduce morbidity [4] and mortality [5], there are challenges associated with its effective use; these include net holes and improper use [6]. The use of currently available artemisinin combination therapy (ACT) is also challenged by emerging resistance to artemisinin on the Thailand-Cambodia border [7].

Vaccines have an excellent, documented, historical record showing its ability to protect human population from diseases; the eradication of smallpox, near elimination of polio, rare cases of measles and yellow fever [8] are all circumstantial evidence of what vaccines can do for public health. An efficacious malaria vaccine will significantly reduce the burden of malaria. The RTS,S malaria vaccine is a pre-erythrocytic malaria vaccine that has been developed by GlaxoSmithKline (GSK) and is being researched with sponsorship from Program for Appropriate Technology in Health/Malaria Vaccine Initiative and GlaxoSmithKline [9]. Clinical trials are ongoing in order to evaluate the safety and efficacy of the vaccine in sub-Saharan Africa, including Ghana. So far, the RTS,S malaria vaccine has been found to be very safe when used among infants and young children [9]. Its protective efficacy against clinical malaria after one year post vaccination among infants and children were about 30% and 50% respectively in phase III trials [9,10]. However, recent efficacy results of the RTS,S malaria vaccine showed a decline in efficacy rate to 16.8% after 4 years of follow up [11]. It may be introduced as part of Expanded Programme on Immunization (EPI) for community use to control malaria following approval by regulatory authorities.

The introduction of new health interventions, including vaccines, has been challenging in some settings. A polio vaccination programme was rejected by community members in northern Nigeria was largely due to perception of religious leaders [12]. In Ghana, community members did not accept a mass deworming programme [13]. In both cases, poor community engagements, including a misunderstanding of the intervention, have been blamed for unsuccessful implementation. Knowledge of community perception of malaria vaccine is therefore required to adequately plan for the introduction of a malaria vaccine if approved by regulatory authorities. A cross-sectional quantitative and qualitative survey was conducted about community perceptions and knowledge of malaria vaccines in RTS,S malaria 47 vaccine trial carried out in the Kintampo area of Ghana. The main findings of the work have been reported [14].

## Methods

A cross-sectional qualitative and quantitative study was carried out in August and September 2007 in the Kintampo North and South districts where phase II RTS,S malaria vaccine trials were being conducted. No efficacy results were available at the time the study was carried out. The districts are located within the forest-savannah transitional ecological zone in the Brong-Ahafo Region of Ghana. Together, the Kintampo North and South districts cover a land surface area of 7,162 sq km, with a resident population of approximately 140,000, based on the Kintampo Health and Demographic Surveillance System (KHDSS) counts [15]; community members are predominantly farmers. Malaria transmission in this part of Ghana is high with an entomological inoculation rate (EIR) of approximately 269 infective bites per person per year and annual average parasite prevalence of 50% children under five years [16]. *Anopheles gambie* and *Anopheles funestus* are identified as effective vectors in the area with similar EIR [17]. Coverage of the EPI in the area is high: vaccination coverage for *Diptheria pertussis,* tetanus*,* poliomyelitis*,* measles*,* yellow fever and all basic immunization in the Brong Ahafo Region of Ghana, of which the study area is part, is over 95% [18]. The majority of inhabitants in this area will likely initiate treatment for all ailments at home then continue to the licensed chemical shops and finally visit a health facility if their health does not improve [19]. There are two public hospitals, three private clinics, three rural clinics, 12 health centres/clinics and 29 community-based health planning and services (CHPS) compounds. These facilities provide health service delivery to both urban communities and deprived rural poor.

### Qualitative methods

#### Focus group discussion

Twelve focus group discussions (FGDs) were conducted among community members in six communities, and 15 in-depth interviews (IDIs) among health professionals and key stakeholders in health in the same communities (Table 1). Communities were purposively selected based on their homogeneities, tribal groupings and geographical location. Selected communities represent half of the total number of communities where the Phase II RTS,S malaria vaccine trial was carried out. One FGD each was conducted in each of the six communities, separately among women or men whose children were participants in this malaria vaccine trial and those whose children were non-participants. Both female and male participants in the FGDs were randomly selected from the KHDSS, which keeps an up-to-date record of the whole resident population. Each FGD session was made up of to 12 participants. It was moderated and recorded for transcription and analysis. In both FGDs and IDIs there was an assessment of the knowledge of vaccines and available community terminologies and names of vaccines, knowledge of EPI vaccines, their attitudes and perceptions on malaria vaccine if found and the availability of any sociocultural beliefs about vaccines. Malaria vaccines were discussed in this survey with the assumption that its efficacy will be similar to that of other EPI vaccines.

**Table 1:** Summary of respondents in in-depth interviews

**Category of respondent**	**Other relevant detail of respondents**	**Total number of interviews**
Medical Officers	Located in public hospital	2
Medical Officer	Located in private hospital	1
Traditional birth attendants (TBAs)	Trained	1
Traditional birth attendants (TBAs)	Untrained	1
Chemical seller	Located in a rural community	1
Chemical seller	Located in an urban community	1
Traditional healers	Located in rural communities	2
Health professionals	Located in rural clinics	2
Methodist Reverend Minister	To represent Protestant churches	1
Catholic catechist	To represent Catholic church	1
Pentecost Reverend Minister	To represent Pentecost Charismatic/Churches	1
Leader of an Islamic sect	To represent Muslims	1
**Total**		**15**

#### Quantitative survey

The household survey involved 466 male and female household heads in the selected communities involved in the qualitative study. The KHDSS was used as the sampling frame. A systematic sampling method was used to select the households in the various communities; half of the respondents were selected from households involved in the malaria vaccine trial and the other half were from households where no child was involved in the malaria vaccine trial. In each compound, only one household was selected to represent the compound. Trained field workers asked questions using a structured questionnaire on knowledge of vaccines and to document available community terminologies and names for vaccines; knowledge of routine EPI vaccines; their attitudes and perceptions on malaria vaccine if one is eventually licensed for routine use and any sociocultural beliefs about vaccines. All respondents consented by signing or thumb printing after the study objectives, procedures, risk and benefits were explained to them, In the case of illiterate respondents, a person designated by the study participant witnessed by signing the consent form in addition to the respondent’s thumb print.

### Data entry cleaning and analyses

All completed quantitative questionnaires were checked and logged in the computer laboratory for data processing and analyses. The quantitative data were analysed using Stata 10 Software (Stata Corp, Texas, USA). Qualitative data from IDIs and FGDs were digitally recorded, transcribed verbatim and checked for completeness and accuracy. Answers to qualitative questions were grouped and categorized using QRS NVIVO version 7 qualitative software. Responses were analysed to identify emerging themes that addressed the objectives. Appropriate quotes that best described the various themes were included as support to the quantitative findings.

### Ethical issues

Ethical approval was sought from the Kintampo Health Research Centre Institutional Ethics Committee. Community entry was by explaining the study’s aims and objectives to key community opinion leaders, followed by all study participants. The study participants were educated on their right to be part of the study or withdraw from it at any point they so wished without suffering any penalty. All participants willingly and individually gave consent; the data collected were kept confidential and with no reference to any study participant.

## Results

### Characteristics of respondents

A total of 466 household heads were involved in the survey after approaching 480 households. A majority (over 74.7%) of respondents were in the age range of 29 and 39 years (Table 2). A majority of the respondents were married; most respondents were farmers (Table 2). Respondents in the FGDs had similar demographic characteristics as those involved in the survey. Those selected for the IDIs were health professionals who had attained at least senior secondary school education or non-professionals with either no formal education or with education up to junior high school level.

**Table 2:** Characteristics of respondents in 2007 household survey

**Characteristics of respondents (N= 466)**	**(%)**
**Age group**	
19-23	1.1
24-28	12.4
29-34	36.1
35-39	38.6
40-44	6.0
45-49	4.3
50-54	1.1
55+	0.4
**Education**	
None	34.3
Primary	18.9
Middle School/Junior Secondary School	38.0
Technical/Commercial/Senior Secondary School	6.2
Post middle/Secretarial	1.5
Post Secondary/Nursing Training	0.4
University/Polytechnic	0.6
**Religion**	
Catholic	16.0
Protestants	13.0
Pentecostal/ Charismatic	23.0
Other Christian	6.9
Moslem	24.0
Traditional	4.7
No Religion	10.3
Other	2.1
**Ethnicity**	
Akan	47.9
Ethnic groups from Northern Ghana	42.3
Other ethnic groups including foreign nationals	9.8
**Occupation**	
Farmer	66.5
Sales	11.8
Skilled manual	9.2
Unskilled manual	8.6
Professional	3.0
Clerical	0.9
**Marital status**	
Married	75.1
Live together	20.6
Widowed	0.2
Divorced	0.2
Separated	1.3
Never married	2.6

### Knowledge of vaccines

Knowledge of vaccines was widespread among participants in the study. Knowledge however, seemed to be skewed towards vaccines given in the form of injections; there was some knowledge of the existence of the oral polio vaccine (OPV), which they admitted is not an injection. Finding a common local name for vaccines that is acceptable and understandable by all respondents was not difficult. Local words like “*ntetee” “paniebo”* exist as terminologies for vaccination. Most respondents knew what vaccines are, as evidenced in the following contributions made by some respondents in both IDIs and FGDs:

“*Vaccines are injections given to children in their childhood so that any disease that has the possibility of attacking children become less severe if even they are attacked.”* (FGD, female E)

*“I know that vaccines are injections given to prevent the occurrence of a disease.”* (FGD, male E)

Participants differentiated vaccines from medicines given as injections in hospitals emphasizing the preventive aspect of vaccines. These observations were made:

*“For vaccines, they are taken to prevent the occurrence of the disease. While those injections that are taken at hospitals, the sickness occurs before one goes for the injections.”* (FGD, male C*)*

### Knowledge of childhood vaccine-prevented diseases

Over 50% of respondents spontaneously mentioned tuberculosis and poliomyelitis vaccines as childhood vaccines (Table 3).

**Table 3:** Knowledge about vaccines in 2007 household survey

**Vaccine type**	**Local name**	**Yes spontaneous n(%)**	**Yes prompted n(%)**	**No n(%)**	**Do not know** n**(%)**
Tuberculosis	“NsamanwaPanie”	171 (36.7)	249 (53.4)	18 (3.9)	28 (6.0)
Poliomyelitis	“MbubuePanie	241 (51.7)	187 (40.1)	10 (2.1)	28 (6.0)
DPT	No local name	61 (13.0)	315 (67.6)	62 (13.3)	28 (6.0)
Measles	NtekyemPanie	244 (52.4)	182 (39.1)	12 (2.6)	28 (6.0)
Hepatitis B	Breboyadeepanie	15 (3.2)	339 (72.7)	84 (18.1)	28 (6.0)
Haemophilus influenza type B	Hwinemuyadee	130 (2.8)	290 (62.2)	135 (29.0)	28 (6.0)
Yellow fever	No local name	162 (34.8)	263 (56.4)	13 (2.8)	28 (6.0)

### Perceptions about preventing malaria through vaccination

There were varied views regarding malaria prevention with vaccines. While some respondents were quite certain that malaria could be prevented through vaccination, others were rather sceptical. This is evidenced in contributions made by respondents in the IDIs and FGDs:

*“In science, there is a saying that you cannot say never. It can happen but I think it will be very difficult. The malaria parasite strains change so rapidly, I think that vaccination could be done but would work in the short term like every three months just like tabs for typhoid fever and the rest. Sometimes you can give it to the person but then it does not last. Its longevity is not there so they would have to come and take it again. Because the malaria strains are so varying and a whole lot of them it have lots of properties and biochemical characteristics, so to get a long-lasting vaccine, it is something we can do but we have to work harder.”* (IDI, health care provider B)

A participant, expressing an opinion about a vaccine’s ability to prevent malaria made the following remarks in an IDI:

*“Well, it looks strange but we are just hoping especially once they say the vaccine is just going through phase II and it is going to phase III. This is because malaria is endemic and any success in that direction is something that everybody is yearning for. At the OPD, the daily OPD register shows that malaria is leading, so many cases of malaria at least 50% or more. The success of this vaccine will even curtail our burden at the OPD”.* (IDI, health care provider A)

When the respondent was asked the reason for describing vaccination to prevent malaria as “strange”, the following was the response:

*“The causative organism is always with us. The mosquito is always with us. Every time we get bitten by a mosquito. I do not know how long, whether the vaccine can stay in the body and produce antibodies that will not let you get malaria once you are vaccinated, just like polio which when your body is exposed to the vaccine you will not get it for your lifetime. This one looks strange because of the causative organism, but it looks impossible, but let’s wait.”* (IDI, health care provider A)

A chemical seller made the following remarks when asked if he believed malaria can be prevented through vaccination. He was a sceptical but made mention of a more multiple approach towards this course to fight malaria*:*

*“Well, it is possible. Chloroquine was a drug which was used to treat malaria when crystalline [*penicillin*] was added but it is now phased out. It [malaria] disturbs people but they cannot complain. We do not know what the Pharmacy Council and the Food and Drugs Board saw about chloroquine and recommend that it should not be used.”* (IDI, chemical seller A)

*“I cannot argue about that. As am saying when the polio vaccine was introduced, the incidence of polio has stopped among children after vaccination. Also with the measles vaccine, a mother who refuses to vaccinate her child is always in trouble whenever the child suffers an attack. So getting a vaccine for malaria is possible. Mosquito nets have been introduced but the incidence of malaria is still high.”* (IDI, chemical seller A)

**Figure 1:**
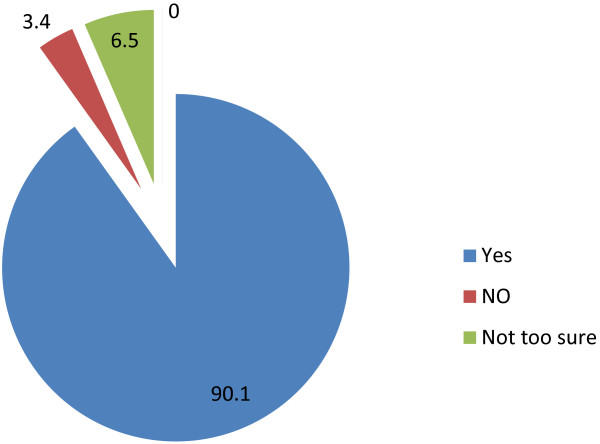
Respondents’ view on whether malaria can be prevented through vaccination in 2007 survey.

The survey showed that a large proportion of respondents think malaria can be prevented through vaccination. Over 90% of the respondents were in this category (Figure 1).

Reasons were assigned for thinking that malaria can be prevented through vaccination; the statement below briefly summarizes the thoughts of participants during a discussion session:

*“An instance is the incident of polio which has been reduced through vaccination. I have the belief that malaria can be eradicated or reduced through vaccination. This can be seen through the research being carried out.”* (IDI, head of a religious group)

### Attitudes towards vaccines for childhood diseases

Respondents’ attitudes about vaccines preventing malaria were quite positive. There was a general quest for vaccines for all types of diseases, especially malaria. Respondents in both FGDs and IDIs mentioned the types of diseases they want their children vaccinated against:

*“Malaria is a sickness that disturbs us a lot. So if children are protected against malaria it will help us. Because there are lots of mosquitoes that bite and cause malaria so that if we prevent the occurrence of malaria it cannot be severe even if there are mosquito bites.”* (FGD, female A*)*

*“Polio, polio disturbs children a lot, it makes them very weak.”* (FGD, female B)

*“With diarrhoea, children vomit and pass out watery stools so if we prevent it, it will be good.”* (FGD, female B)

### Vaccines or drugs for malaria control

**Figure 2:**
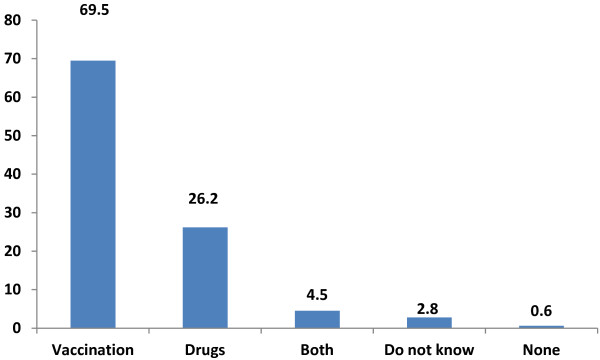
Preference between vaccines and drugs for malaria in 2007 survey.

As part of learning their attitudes towards vaccines for malaria, respondents were asked whether they preferred vaccines or drugs or both for malaria; 65.9% of respondents preferred vaccines to drugs for malaria control while 26.2% preferred drugs to vaccines. Few respondents had no preference for vaccines or drugs (Figure 2).

Responses from both male and female participants on whether they will allow their children to be vaccinated against malaria, assuming a malaria vaccine is found, were very insightful:

*“I will agree: I have no drugs to cure my children. So if the government says a disease is about to break out so I should vaccinate my child, what can I say?”* (FGD, female E)

*“Malaria is the number one killer diseases among children so if it is going to be prevented, then I think everyone would be happy to include his/her child.”* (FGD, male A)

Diseases that respondents wanted their children vaccinated against were confirmed in the survey (Table 4). These were affirmed by some of the responses in the FGDs, thus emphasizing how generalized some of the contributions made by participants in the FGDs could be when it came to the diseases participants will or will not want their children to be vaccinated against (Table 4).

**Table 4:** Diseases that respondents will want their children vaccinated against (2007 household survey)

**Disease type**	**Local name**	**Yes spontaneous n (%)**	**Yes prompted n(%)**	**No n(%)**	**Do not know** n(%)
Malaria	“Atiridie”	361 (77.6)	75 (16.0)	3 (0.6)	27 (5.8)
“Hot body”	“Ahoohyee”	175 (37.6)	242 (51.9)	21 (4.5)	27 (5.8)
Cough or other respiratory problems	“Ewa”	112 (24.0)	275 (59.0)	52 (11.2)	27 (5.8)
Diarrhoea	“Ayamtuo”	85 (18.2)	282 (60.5)	72 (15.5)	27 (5.8)
Childhood rashes	“Nsawansawa”	66 (12.9)	345 (74.0)	34 (7.3)	27 (5.8)
Measles	“Ntenkyem”	240 (51.5)	188 (40.3)	11 (2.4)	27 (5.8)
Abdominal pains	“Yafunuyadee”	38 (8.2)	330 (70.8)	71 (15.2)	27 (5.8)
Tuberculosis	“Nsamanwa”	183 (39.2)	236 (50.6)	20 (4.3)	27 (5.8)
Convulsion	“Esoro”	111 (23.8)	288 (61.8)	40 (8.6)	27 (5.8)

Among health care providers and community religious leaders it was evident that vaccinating children against malaria will be a major breakthrough in science and accepting it will not be very challenging at the community level:

*“I will readily recommend that because malaria in children under five is fatal. Cerebral malaria for instance causes lots of problems. In children under-five, because of their immune system, when they get malaria it is fatal because of its added complications. Should we make a head way with this vaccine trial, I think all children, most especially, children under five should be vaccinated.”* (IDI, health care provider)

*“Readily yes or even should I say a big yes. You see, these children are exposed to lots of filth and as you can see lots of weeds around that serve as breading grounds for mosquitoes. So getting a vaccine to prevent the effects of these mosquito bites will be a good thing. I will recommend it.”* (IDI, head of a religious group)

### Beliefs and cultural practices about vaccines

The success of disease control programmes to a large extent depends on the beliefs and cultural practices of the people who are directly involved in the programme and this shapes their behaviour and consequently their decision-making processes. Though beliefs and cultural practices that will prevent parents from vaccinating their children were not mentioned, some IDI sessions revealed some interesting points are quoted below:

*“……..somewhere in the north, when a child is born it is not brought out until after one month or so. So if we are looking at a vaccine targeting children of four weeks or less, that can possibly be a barrier to such children getting access to the vaccine. Another one is the social barrier where women cannot take decision about their children.”* (IDI, health care provider)

*“As a Catechist who is in charge of organizing the congregation here, I always advise women who take their children to “weighing” [*child welfare clinics*].” In the olden days herbs were used to treat diseases but now it is a thing of the past. We also advise against the belief that sickness is caused by gods. For now, it is only medication that is given which in the name of Jesus we also pray to support I don’t know of any such beliefs. In the beginning of the trial [*RTSS malaria vaccine trial*], some people from the north living here did not understand that there is the need send the child for immunization and weighing.”* (IDI, religious leader)

*“There is nothing like that [*cultural beliefs*] if you should follow these things all your children will die.”* (FGD, male C)

Some religious dimension was given by a participant when asked if he will recommend vaccination for malaria:

*“I know that God has poured his grace on a group of people who are working through the Holy Spirit. They are doctors. As the Bishop or any church member goes to the hospital when sick, if a vaccine for malaria is found I will not prevent people or any of my church members from sending their children for vaccination.”* (IDI, head of a religious group*)*

Among the Muslim participants, the situation about recommending vaccines for malaria was not very different. A participant had this to say:

*“Even the prophet Mohammed (‘peace be on him’) implored his followers to do two important things: learning very well so that you can worship your God; learning about protecting yourself from falling sick. It will have been welcome news to be able to protect ourselves against diseases. Even God approves of that. People should actually treat themselves when they are sick. It is also mentioned in the Quoran that people should protect themselves from falling sick. It is good to “defend” yourself from sickness. This is better than allowing the disease to attack you before you treat it. Yes, the Quoran does not forbid vaccination. Quoran is totally in support of prevention. I will readily recommend, if even it means talking about it in the various mosques that are under my jurisdiction. I will even mobilize other Imams working under me, so we can educate people.”* (IDI, head of a religious group)

### The call for well-researched studies on a malaria vaccine

Community members called for research as the basis for acceptance or otherwise of a malaria vaccine, if one is found. The following observation was made in this regard:

*“If research proves that vaccines can be used, then you can use it. If research does not prove that vaccines can prevent that disease then you do not have to use it. So every disease that research proves to be preventable with vaccines you can vaccinate to prevent it”*. (FGD, male E)

*“We want vaccines for communicable diseases but not for other diseases that we can buy drugs at home to cure. For instance, when you suffer from headache, if you find a drug vendor around, you can buy some drugs. Also when you suffer from stomach aches, you can equally buy drugs from vendors. These do not require vaccines as they are not communicable.”* (FGD, male E)

### Some concerns expressed about the trial

Some participants expressed worry about the volume and frequency of blood taken from their children for laboratory analysis and delays at the trial facilities. They were however quick to add that they knew the processes were legitimate for the study as indicated below:

*My child is not in the trial but because of this blood draw someone withdrew the child from the study. So I went to the person and explained him that in this trial, it is difficult to even lose a child and if even one child dies it will be a drawback to the work . People may think it is because of the blood draw, the blood is only used to do some investigation, it is not that it is going to be given to any elderly person or it is going to be sold.”* (FGD, male C)

*“I think the blood drawn is for the test of the child’s own health. For example, when they were enrolling the children for the study, my child was screened and because he had some health problems as the result of the blood test stated, I was made aware that because of that my child was not going to be enrolled into the study and I agreed.”* (FGD, female E)

## Discussion

Understanding the local context of diseases and the communities’ perceptions of the experimental interventions serves as prerequisite for how the intervention will be accepted and utilized when it becomes a policy that has to be implemented as a programme. Several studies have documented the need and how best to educate communities [20-23]. This study’s findings, which are similar to studies documented on the need to understand the local context of diseases, have benefited from the experiences of such studies and has identified findings as an important step in ensuring the malaria vaccine will be acceptable to communities where this was tested and serve as the springboard for integrating the vaccine into other communities that did not necessarily participate in the trials.

It is significant to understand how community members perceive a malaria vaccine in order to inform the country’s malaria control managers and officials responsible for the EPI for planning purposes.

Our results are similar to that found in Kenya [20]. Knowledge of childhood vaccinations was widespread among respondents. Local names were provided for them, and this could have some positive effects on childhood immunization programmes. Caregivers will at given times be able state which immunizations their children had received and those that they have not received. However in some parts of Mozambique, caregivers mentioned vaccines that do not currently exist [24], and this could have some negative effect on childhood immunization and overall child care.

The perception of a malaria vaccine as a preventive tool was similar to that found in a study in the south coast and the Busia districts of Kenya; respondents were however more guarded on the possibility of a malaria vaccine [20].

The role of religious beliefs in shaping perceptions and consequently decisions need not be over emphasized. Religious beliefs have the potential of diluting community perceptions on a given phenomenon. The boycott of the polio vaccination programme in some parts of Nigeria was partly because of the belief that the vaccine could be contaminated with anti-fertility, HIV and cancerous agents. This was mainly a concern held by Muslim clerics in Muslim-dominated states and by the Supreme Council for Sharia in Nigeria (SCSN) [12]. Unlike the Nigerian incident, this study revealed a contrary position; religious beliefs that could serve as a potential barrier to the acceptance of a malaria vaccine were virtually unknown in the study area, not even among religious leaders. Religious leaders could therefore be a conduit for a mass introduction of malaria vaccines. In this study there was a community belief children should not be seen until they are more than a month old. In a recent study in Mozambique it was reported that a common taboo among some communities was that a newborn should have limited exposure to adults with “hot bodies” and “hot blood” [24]. The belief about exposure of newborns was not limited to adults only, as reported in the Mozambique study. This belief however is unlikely to affect the administration and coverage of new vaccines, such as malaria vaccines, since some known childhood immunizations such as OPV are given within the first month of life and their coverages are high. Women who deliver carry their children to immunization points for such immunization schedules.

The contribution of the environment is key when chronicling factors that contribute to the breeding of mosquitoes. Recent evidence in Tanzania showed that most vector control measures target life-threatening mosquitoes through the use of chemicals belonging to the pyrethroid class; however resistance of mosquito populations to the pyrethroid class of chemicals is increasing dramatically [23]. Community members recognized the role the environment plays in the breeding of mosquitoes. They recognized challenges to malaria vector control, especially when there is growing resistance to available insecticides by mosquitoes. They will therefore welcome a vaccine as one of the ways of surmounting this problem.

The popularity of vaccines against tuberculosis, measles and poliomyelitis compared to hepatitis B and *Haemophilus influenza* type B vaccines is because the former vaccines have been in existence over several years compared to the latter. Similar experience was found among Hong Kong Chinese adults, where insufficient awareness of hepatitis B infection was observed. The poor knowledge of this infection may have resulted from the poor knowledge of the universal neonatal and adult hepatitis B vaccination [25]. Health managers and professionals should be aware of the results of this study and revamp their effort towards education on the relatively new vaccines.

The study was conducted at the time when malaria efficacy results were unavailable. The knowledge and perceptions of malaria vaccines were therefore assessed with the assumption that its efficacy will be as high as that of other childhood vaccines. It is likely that the perception of malaria vaccines and their acceptability would be different if respondents were aware of the current relatively lower efficacy results of malaria vaccines compared to other childhood vaccines [11]. It is therefore important for further studies to be conducted in the context of current malaria vaccine efficacy results.

### Limitation

In light of the high illiteracy levels of respondents, it was not possible to conduct interviews in English. FGDs and some IDIs were conducted in “Akan” (the main local language). Thus, some questions had to be reframed when the respondents had difficulty in understanding. Some respondents lacked in-depth understanding of the local language and may not have satisfactorily expressed themselves. The responses may not completely reflect the perceptions of communities where none of the vaccine trial(s) have taken place. It will be useful to carry out similar interviews in communities outside the Kintampo study area in order to document the views of such communities.

## Conclusions

Most respondents will want their children vaccinated against malaria as part of routine vaccinations. Knowledge of the EPI vaccines was common among the study population. They could distinguish easily between those that are given as injection and those that are given through other forms. A large number of respondents in the study wish their children to be vaccinated for all the known diseases covered by the EPI. Comparatively greater number of respondents preferred vaccines to drugs for malaria control assuming the vaccine is highly efficacious. Beliefs and cultural practices that will serve as barriers to the acceptance of a malaria vaccine were virtually unknown.

## Competing interests

The authors declare that they have no competing interests.

## Authors’ contributions

LGF conceptualized the idea and was responsible for the design of data collection tools, supervision of data collection, data analyses, management and writing up as the principal investigator. KPA and SOA contributed to the design of data collection tools, data analyses management and write up. DSD and KAS contributed to the design of the data collection tools and analyses as supervisors of the MPhil (Sociology) Thesis. LGF drafted the initial paper with extensive review from KPA and SOA. DSD, KAS and TSL reviewed the manuscript at an advanced stage. All approved the current version of the manuscript.
